# Development of a prospective cohort of HIV Exposed Sero-Negative (HESN) individuals in Jos Nigeria

**DOI:** 10.1186/s12879-016-1649-1

**Published:** 2016-07-22

**Authors:** Sophia Osawe, Evaezi Okpokoro, Ruth Datiri, Grace Choji, Felicia Okolo, Pam Datong, Alash’le Abimiku

**Affiliations:** Plateau State Human Virology Research Centre (PLASVIREC), Jos, Nigeria; Institute of Human Virology (IHVN), Abuja, Nigeria; Institute of Human Virology, School of Medicine, University of Maryland, Baltimore, USA

**Keywords:** HIV, HESN, Couples, Vaccine, Incidence, Nigeria

## Abstract

**Background:**

HIV/AIDS continues to be a global health problem. With currently no cure, it is critical to get an effective vaccine to add to the arsenal of prevention and treatment tools. HIV Exposed Sero-Negative (HESN) individuals were enrolled and followed for 2 years.

**Methods:**

A prospective observational cohort study to enroll HESN volunteers and their partners was developed with a 2-year follow up. This was a vaccine preparedness study and designed as a Phase IIb trial. We provided counseling, lab testing and conducted medical examinations for all enrollees.

**Results:**

A total of 534 HESN were enrolled with 48 % (256) females and 52 % (278) males, a mean age of 37 ± 9 years. Three female HESN enrollees seroconverted giving this cohort a HIV incidence rate [95 % coefficient interval (CI)] of 3.2 (2.3–4.2) per 100,000 person-months of observation. Baseline analysis showed that female HESN are 24 % more likely to have their spouse consistently use condoms (RR 1.24; *p* = 0.04); 16 % more likely to have HIV+ partners with detectable viral load (RR 1.16, *p* = 0.03) and 28 % more likely that their HIV+ partners has a CD4 count less than 350cells/μl (RR 1.28, *p* = 0.03) when compared to male HESN.

**Conclusions:**

Our findings suggest that female HESN are more at risk of HIV acquisition due the low CD4 counts and detectable viral load among their HIV+ spouses. Moreover, we provide additional information on incidence and risk factors among naturally exposed persons, which might impact biomedical prevention research and immune responses to HIV vaccines.

## Background

HIV/AIDS remains a global health problem. At the end of 2012, over 35 million were infected and living with the virus with an estimated 2.3 million new infections. Sub-Saharan Africa bears over half of the burden of HIV infections [1]. Nigeria is the most populous country in Africa with an estimated population of a little over 168 million [2] and has the second highest burden of HIV in the world after South Africa [3]. The HIV prevalence in Nigeria is 4.1 % based on antenatal sentinel surveys carried out bi-annually [4]. Despite this, no HIV vaccine trial has been conducted in Nigeria although it participated in a microbiocide trial that was stopped due to a recorded low HIV incidence [5].

The government of Nigeria initiated antiretroviral treatment programs in 2002 [6]. However, there were setbacks with this plan as a result of drug stock outs [7]. In 2004, the US President’s Emergency Plan For AIDS Relief (PEPFAR) provided antiretroviral treatment in Nigeria [8] and along with Global Fund has contributed to the new approach for the use of antiretroviral drugs (ARVs) to prevent HIV transmission [9]. The HPTN 052 trial showed that early commencement of ARV at higher CD4 count significantly reduces HIV transmission among HIV sero-discordant couples [10]. Despite this, a significant number of new HIV transmissions still occur among heterosexual sero-discordant couples in Sub Saharan Africa [12–14] and majority occur in partners unaware of their sero-discordant status [12].

Discordant couples remain a medium risk group for HIV transmission [14] with continuous natural exposure and higher recorded retention rates, making this group suitable for HIV biomedical prevention studies including vaccine trials and/or for understanding immune responses in HIV uninfected exposed individual who subsequently get vaccinated. Developing a cohort of HIV Exposed Sero-Negative (HESN) individuals in a resource limited setting like Nigeria will help prepare grounds for a future HIV prevention trials like HIV vaccine trials. As Nigeria has never had a HIV vaccine trial it was important for us to have a demonstration phase by developing and following up a cohort. The gaps surrounded capacity building for HIV vaccine clinical sites and national regulatory bodies that would supervise a vaccine trial in the country. We also wanted to develop a well-characterized cohort to document HIV incidence, Risk factors, circulating subtypes, retention rates and willingness to participate in a future vaccine trial, all crucial data for future vaccine trials [15]. Our community engagement arm created awareness in the community on HIV biomedical prevention tools including microbicides.

## Methods

### Study design, setting and ethics

A prospective observational cohort study to enroll HESN partners in an established sero-discordant relationship with a 2-year follow up was developed at the Plateau State Human Virology Research Centre (PLASVIREC), a research facility in Jos, Nigeria. The study was conducted from October 2011 through December 2015. Suitable volunteers were invited through the counseling unit of PLASVIREC, community referrals, outreaches done by Civil Society Organizations (CSOs) and support groups at PEPFAR care and treatment facilities to the study sites for recruitment. To continue to support couple counseling, we also offered CD4 and viral load estimation to the HIV positive partner of the HESN enrollees receiving care at PEPFAR supported treatment centers.

Relevant ethical approvals were obtained from three institutional review boards: the site IRB committee (Plateau State Specialist Hospital, Jos); the National Health Ethics Committee (NHREC); and the international collaborating institution (University of Maryland, School of Medicine) IRB. Volunteers were screened at PLASVIREC, the research site and four other satellite sites clustered around in Plateau (COCIN Hospital Rehabilitation Center Mangu- CHRCM) and neighboring Nassarawa (Dalhatu Araf Specialist Hospital- DASH and Federal Medical Center Keffi -FMCK) and Bauchi states (Abubakar Tafawa Balewa Univesity Teaching Hospital- ATBUTH). The four satellite sites were sites tertiary PEPFAR sites trained and supervised by PLASVIREC laboratory and clinical staff. These sites are care and treatment centers with a number of dedicated staff and well-equipped laboratories that handle specimen processing and short-term storage. Consent forms used were approved by all IRBs and every volunteer provided a written consent before enrollment into the study.

### Study procedures

#### Screening and enrollment

Eligibility criteria were used to screen healthy HESN living in a sero-discordant relationship before consenting and enrollment into the study. These criteria included: being HIV negative, in a stable discordant relationship for at least 3 months, between 18 and 65 years old, being able to provide informed consent, willing to continue with follow up study visits and provide locator information.

#### Study visits and processes

The study visits mimicked those of a phase IIb vaccine trial with a total of 11 visits spread across 2 years, at baseline, month 1, month 2, month 3 and 3-month intervals for the other 7 visits as part of preparing the site and cohort for HIV vaccine and biomedical prevention clinical trial. HESN enrollees were given study cards with their enrollment numbers and the next visit date as a reminder. Pre-test and post-test couples HIV voluntary counseling and testing (CVCT) and risk reduction counseling were offered at screening, enrollment and follow up visits. Free condoms were distributed to all volunteers and counselors demonstrated the proper use of condoms with penile models. Counselors and study nurses collected sexual behavioral data at every visit including condom use. All HESN enrollees were tested to establish their HIV status at enrollment and a standardized questionnaire was used to collect demographic and medical history with full medical examinations conducted by trained study clinicians. Venous blood draws were done using one 10 ml EDTA tube and one 9 ml plain (SST) tube at each of the eleven visits from study enrollees for a number of laboratory assays including HIV syphilis and hepatitis C serology, pregnancy tests for females, complete blood counts (CBC) and liver and kidney function tests. To maintain standard processes, all study procedures were performed at PLASVIREC and satellite sites by the same trained research team.

The counselors kept an electronic diary to track all follow up visits of the enrollees. Phone calls were proactively made to remind volunteers of their next appointment at least two days prior to their visit. Enrollees that missed their appointment were reached through phones and if not successful, a tracking team visited their homes with their approval. Failure of study enrollees to come back for follow up visits over a period of three months or of the trackers to trace an enrollee, led to termination and exit from the study. Reasons for termination were recorded as reported elsewhere [Osawe et al. [16]]. Retention rates were calculated throughout the duration of the study [Okpokoro et al. [17]].

### Laboratory tests

#### HIV Exposed Sero-Negative study enrollees

HIV rapid test was performed for all HESN enrollees at each visit based on the National testing algorithm using Determine (Abott Laboratories, Japan) to screen and a second test using Unigold (Trinity Biotech, USA) on samples that screen positive. For discordant HIV result, a tiebreaker test was done with StatPak (Chembio Diagnostic Systems, USA). ELISA kits were used to test serum samples for Syphilis (Lifemed Diagnostic products, Canada) and Hepatitis C (Biotec Laboratories Products, UK) antibodies. Complete blood counts were performed on a Sysmex KX2N1(Sysmex Corporation) platform while VITROS 350 (Johnson-Johnson Orthodiagnostics) was the platform used to run chemistry assays to test for liver and kidney function tests.

#### HIV Sero-positive partners of HESN study enrollees

CD4 counts were performed using Cyflow SL-3 (PARTEC, Germany) and viral loads were done using CAP-CTM HIV-1 version 2.0, with a detection range of 20–10,000,000 copies/ml (ROCHE, Germany). Results were given to their service provider.

### Data collection and statistical analysis

Standardized case report forms (CRFs) were used for data collection. For the sample size of this study a convenient size of 500 HESNs was planned for the study to document the capacity of the study site to recruit, enroll and retain HIV negative persons into a study involving 24 months of complex trial visits conducted at a Good Clinical Practice (GCP) level of documentation and adverse event reporting to the sponsor, regulatory agency, and ethics committee. A total of 534 volunteers were enrolled to compensate for the anticipated 7 % that would be loss to follow up during a follow up of 2 years. Considering an anticipated 0.5 % incidence rate, this number would provide enough transmission events to document the social and viral dynamics of transmission in this relatively normal population. Socio-demographic data were summarized at baseline and presented in absolute numbers and percentages. Student *t*-test was used to compare the mean of continuous variable while fisher’s exact chi square test was used to compare proportions. We stratified our data based on gender and calculated the risk ratio. Variables compared between male and female HESN include: condom use, number of sex partners, self reported STIs, syphilis and Hepatitis C laboratory test results, HIV seropositive spouse/partner viral loads and CD4 counts. We compared female HESN enrollees to male HESN enrollees (i.e., using males as the reference or comparative group). The cumulative risk (or incidence) of critical variables (i.e., HIV cumulative risk) and incidence rates was also calculated. Significant level was set at p ≤ 0.05. For the HIV sero-positive partners of the HESN study enrollees, history of ART, reports of any sexual transmitted disease and tuberculosis were obtained from their medical records following IRB approval. Vetted forms were entered into MS Access database and analyzed in STATA 12 (Stata Corp, College Station, Texas, USA).

## Results

### HESN enrollees

A total of 534 HESN volunteers who were in an established sero-discordant relationship, were enrolled into this cohort between October 2011 and December 2012. We enrolled 203 volunteers at PLASVIREC, 137 at DASH, 82 at ATBUTH, 39 at FMCK and 73 at CHRCM. The cohort had an almost equal distribution between males and females, 256 (47.9 %) females and 278 (52.1 %) males with a mean age of 37 ± 9 years (range 19–65 years) (Table 1). Other baseline demographic characteristics of the HESN study enrollees showed 99 % are married, out of which 92 % reported being in a monogamous marriage, 45 % were 31–40 years old, 33 % had no formal education and the predominant religion among enrollees was Christianity (76 %).

**Table 1:** Socio-demographic characteristics of HESN study enrollees

Socio-demographic characteristics^a^	*N* = 534^b^
Gender	
Male	278 (52)
Female	256 (48)
Age (years)	
Mean ± SD	37 ± 9
Range	19–65
Age group	
≤ 30	136 (25)
31–40	237 (45)
41–50	116 (22)
> 50	45 (8)
Marital Status	
Married	529 (99)
In a relationship/cohabiting	5 (1)
Religion	
Christianity	407 (76)
Islam	127 (24)
Educational status	
Non formal	59 (11)
Primary	138 (26)
Secondary	159 (30)
Tertiary	178 (33)
Employment status	
Employed	252 (47)
Unemployed	281 (53)

^a^SD, Standard deviation; N, total number

^b^All values are n (%) unless specified

At baseline, we found that 40 % (215/534) of HESN use male condoms consistently, 30 % (158) use condoms occasionally and 30 % (161) never use condoms. In addition, 8 % (42/534) of HESN enrollees have 2 or more sexual partners . As shown in Table 2, risk factors for HIV acquisition differ by gender and so were stratified by accordingly. We found that females HESN enrollees were 24 % more likely to have their partners use condom consistently (RR 1.24, CI [1.01–1.54], *p* = 0.04) when compared to male HESN. This is probably due to availability and affordability of male condoms. Additionally female HESN were 78 % less likely to have 2 or more sexual partners (RR 0.22, CI [0.09–0.48], *p* < 0.0001) and 64 % less likely to report history of STI (RR 0.36, CI [0.19–0.7], *p* < 0.0001). On the contrary, female HESN were 16 and 28 % more likely to have HIV positive partners (i.e., male spouses) with detectable viral load and CD4 count less than 350cells/μl respectively (*p* = 0.03) (Table 2). In other words, the male partners of the female HESN enrollees were sicker and more likely to transmit the virus to their female counterpart. This was confirmed by the fact that all 3 HESN that seroconverted during the follow up period of this study were females giving a HIV incidence of 3.2 CI [2.3–4.2] per 100,000 person-months of observation. Nine HESN enrollees were sero-positive for syphilis at baseline giving a prevalence of 1.7 % (9/534) and 40 were sero-positive for hepatitis C virus (HCV) infection giving a prevalence of 7.5 % (40/534). During follow up visits, we found 2 new cases of syphilis and 19 new cases of HCV giving a cumulative incidence of 0.4 % (2/525) and 3.8 % (19/494) respectively. The mean follow up period of the HESN sero-converters was 2.6 months.

**Table 2:** Baseline risk factors for HIV acquisition in the HESN cohort stratified by gender

	*N* = 278^a^	*N* = 256	Risk Ratio (95 % CI)^c^	P-value
	Males^b^	Females		
Condom use			1.24 (1.01–1.54)	0.04
Always	100 (36)	115 (45)		
Sometimes/Never	178 (64)	141 (55)		
Sex partners			0.22 (0.09–0.48)	<0.0001
< 2	243 (87)	249 (97)		
≥ 2	35 (13)	7 (3)		
Reported STIs prior to enrollment			0.36 (0.19–0.7)	<0.0001
No	245 (88)	245 (96)		
Yes	33 (12)	11 (4)		
Syphilis			2.18 (0.55–8.66)	0.32
No	275 (99)	248 (98)		
Yes	3 (1)	6 (2)		
Hepatitis C virus (*N* = 524)			0.89 (0.49–1.63)	0.72
No	252 (92)	232 (93)		
Yes	22 (8)	18 (7)		
HIV+ partners of HESN enrollee				
	HIV+ positive (female spouse)^d^	HIV+ positive (Male spouse)		
Detectable partner Viral load (*N* = 383)	*N* = 214	*N* = 169	1.16 (1.02–1.32)	0.03
No (≤20)	76 (35)	41 (25)		
Yes (>20)	140 (65)	126 (75)		
Partner CD4 count (*N* = 375)	*N* = 212	*N* = 163	1.28 (1.03–1.6)	0.03
> 350 cells/μl	126 (59)	78 (48)		
≤ 350 cells/μl	86 (41)	85 (52)		

^a^All values are n (%) unless specified

^b^Male is the reference group

^c^CI, coefficient interval

^d^Female spouse is the reference group

### HIV+ partners of HESN study enrollees

**Fig. 1:**
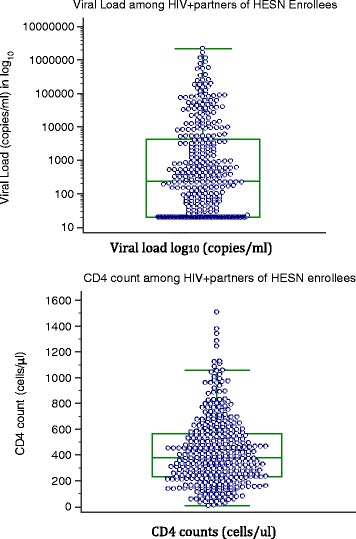
Box Plots Showing CD4 Count And Viral Load In The HIV+ Partners Of The Study Enrollee. Viral loads were transformed to log_10_ and reported in copies/ml. CD4 counts are reported in cells/μL

We found that among HIV+ partners of HESN study enrollees, 85 % were already on antiretroviral treatment provided by the PEPFAR program. The mean CD4 count was 424.5 (±263cell/ μl) with a range of 6–1505 cells/ μl. A significant number (34 %) had CD4 counts <350 cells/μl, suggesting late access to care which is common in resource limited countries such as Nigeria. We also found that about 70 % (266/383) tested had detectable viral loads despite being on HAART for an average of 5 years; with 19 % (72/383) with viral loads of ≥10,000 copies/ml out of which 58 % (42/72) were female. Mean viral load of the HIV sero-positive partners was 38,223copies/ml with a range of <20 - 2,186,199 copies/ml (20 copies/ml is the cut-off point for detectable viral load). Figure 1 shows the distribution of CD4 counts and viral loads (converted to log10) among HIV positive partners of the HESN enrollees.

## Discussion

Our study successfully developed a prospective HESN cohort and well-trained research team that was able to build capacity at the other study satellite sites. The research team focused on collecting complete data using the standardized CRFs across all the study sites and engaging in continuous tracking activities that helped the study to maintain high retention rates as described elsewhere [17], a critical component of vaccine and prevention trials [18]. Couples and risk reduction counseling were provided at all study sites and at every visit for the HESN study enrollees and their partners. Our study shows that despite the availability of treatment programs, HESN in a sero-discordant relationship continue to be at risk of being infected by their HIV sero-positive partners in resource-limited settings making this cohort relevant to HIV biomedical prevention studies. We showed that 19 % of the tested HIV sero-positive partners had viral loads >10,000 viral load and male condom use in a significant number of the cohort is inconsistent (Table 2). Three of the enrollees that seroconverted in our study reported inconsistent use of condoms and had partners with detectable viral loads despite being on antiretroviral therapy probably due to suboptimal ARV adherence leading to resistance, which is common in our setting. All 3 that seroconverted were women whose partners were quite sick with low CD4 counts further underscoring the additional risks that women face. Among the HIV+ partners, 58 % who had high viral loads (>10,000cells/ml) were females. We are not certain why this is the case but it may have to do with adherence challenges due to increased stigmatization against women in such cultural settings. Women are also more likely to share their ARVs with other sick persons.

Although couple counseling, risk reduction counseling and free condoms were given at every follow up visit; a significant number of enrollees consistently reported never using condoms or inconsistent use. This is problematic and similar to the experience reported by two studies in Uganda [19, 20]; since as reported in other studies, HIV Viral Load is a risk factor for HIV transmission [21, 22]. Allen et al. reported significant improvements in condom use through continuous risk reduction counseling [23] so we adopted this in our cohort and were able to show improvement in condom use, though not statistically significant (data not shown). Reasons for poor condom use among this cohort included reduced sexual pleasure. Inconsistent condom use was higher among male HESN enrollees, which is not surprising in the African setting where the male is the head of the family and makes the decisions, while women are expected to be submissive to the spouse. Moreover female condoms were not easily available and amongst married couples, there is the added cultural need to bear children as evident in 22 females who became pregnant during the course of our study similar to data from a similar cohort in Ugandan [19].

In our study more women tested positive for syphilis positive and had history of genital discharge prior to enrollment (i.e., at baseline). This may have placed them at more risk for HIV acquisition as evidence by all 3 females who seroconverted within four months of follow up despite the risk reduction and couples counseling offered indicating that such interventions would need to start at an early age for both sexes. This agrees with earlier studies that show that women are more at risk of getting infected by their male partners [11, 26]. However, our finding was not statistically significant due to the small numbers sero-converters in our cohort. Since over 90 % of Nigerian men are circumcised, circumcision was not considered as a risk factor in our cohort unlike other East African cohorts.

At the moment, the recorded HIV incidence in our cohort is 3.2 % per 100 person-months of observation, which is lower than that desirable for HIV vaccine efficacy trials [24, 25]. It is also lower than that reported for a HIV vaccine preparedness cohort in Uganda and India [19, 24]. Our study was performed at a time when ARVs were widely available in Nigeria and this would obviously affect transmission in this cohort. Nevertheless, as we showed, adherence and viral suppression continue to be a significant challenge as ARVs is scaled up to all that require it. Our study provides valuable insights into this cohort as HIV biomedical prevention studies are being ramped up globally and in Nigeria and reveals that long term transmission possibilities in this group of individuals is still were the viral load is not suppressed as reported in other studies [27].

There were however limitations in this study. Recruitment was based on referrals and walk in HIV testing volunteers and some data collected on risk were based on self-reporting. With self- reported data there are limitations with recall bias and therefore risk may be under reported or over reported.

## Conclusion

In conclusion, our study has established a fairly healthy HESN prospective cohort; an important natural adult model to study HIV transmission, immune response in HIV exposed adults and effectiveness of HIV prevention measures including the testing of candidate HIV vaccines. Our study is the first of its kind in Nigeria and has established HIV incidence in the presence of ART and STI prevalence among HESN in an established sero-discordant relationship. This will help guide the conduct and interpretation of future HIV prevention studies among this cohort including a future HIV vaccine trial.

## Abbreviations

ART, antiretrovial treatment; ARVs, antiretrovirals; CBC, complete blood count; CD, cluster of differentiation; CI, coefficient interval; CSOs, Civil Society Organizations; CVCT, couples voluntary counseling and testing; HESN, HIV Exposed Sero-Negative; HIV, human immunodeficiency virus; IRB, Institutional Review Board; NHREC, National Health Research Ethics Committee; PEPFAR, Presidents Emergency Plan for AIDS Relief
